# [Retracted] MicroRNA‑451a prevents cutaneous squamous cell carcinoma progression via the 3‑phosphoinositide‑dependent protein kinase‑1‑mediated PI3K/AKT signaling pathway

**DOI:** 10.3892/etm.2024.12568

**Published:** 2024-05-13

**Authors:** Jixing Fu, Jianhua Zhao, Huamin Zhang, Xiaoli Fan, Wenjun Geng, Shaohua Qiao

Exp Ther Med 21:116, 2021; DOI: 10.3892/etm.2020.9548

Following the publication of this paper, it was drawn to the Editor’s attention by a concerned reader that certain of the cell proliferation assay data shown in Fig. 2B on p. 5 were strikingly similar to data appearing in different form in Fig. 2A of another article written by different authors at different research institutes, which had already been submitted for publication at the time of this paper’s receipt at the *Experimental and Therapeutic Medicine* Editorial Office [Duan R, Li C, Wang F, Han F and Zhu L: The long noncoding RNA ZFAS1 potentiates the development of hepatocellular carcinoma via the microRNA-624/MDK/ERK/JNK/P38 signaling pathway. Onco Targets Ther 19: 4432-4444, 2020].

Owing to the fact that the contentious data in the above article had already been submitted for publication prior to its submission to *Experimental and Therapeutic Medicine,* the Editor has decided that this paper should be retracted from the Journal. The authors were asked for an explanation to account for these concerns, but the Editorial Office did not receive a reply. The Editor apologizes to the readership for any inconvenience caused.

